# Commentary: A crisis in comparative psychology: where have all the undergraduates gone?

**DOI:** 10.3389/fpsyg.2015.01589

**Published:** 2015-10-14

**Authors:** Neil McMillan, Christopher B. Sturdy

**Affiliations:** ^1^Department of Psychology, University of AlbertaEdmonton, AB, Canada; ^2^Neuroscience and Mental Health Institute, University of AlbertaEdmonton, AB, Canada

**Keywords:** comparative psychology, comparative cognition, recruitment, undergraduates, graduate programs, education

Consider our surprise to suddenly find out that, as researchers in the field of comparative cognition, we are in fact not comparative psychologists. We admit to not having consistently referred to ourselves specifically as comparative psychologists, so perhaps we have not lost a definitive component of our identities, but in turn it feels like waking up one day to find one's pinky fingers have been surreptitiously removed and to spend the rest of the day continually realizing that they had in fact previously been there all along.

## What's in a name?

Core to Abramson's (2015) paper is the assumption that comparative psychology is a unique discipline to all others. Abramson gives little indication of what does and does not constitute comparative psychology, except to present his belief that it cannot be wholly subsumed by integrative biology, evolutionary psychology, or comparative cognition. However, this only seems a lesser version of the problem faced by experimental psychologists for decades: is there a psychology that we all study? We would not all necessarily classify ourselves social psychologists, cognitive neuroscientists, or applied behavior analysts. And we are often loath to introduce ourselves to other scientists and laypeople as *psychologists*, for fear of the ensuing stereotypes too diverse and painful to discuss here. Does that mean none of us *are* psychologists, that none of us study psychology?

The doom and gloom surrounding the future of comparative psychology seems mostly constrained to the specific use of the term. Here the supposed death rattle of the discipline is based on a dearth of results for narrow searches of a single term, “comparative psychology,” as though a scientific field lives or dies on the number of times its nebulous moniker is uttered. We researchers interested in questions about animal behavioral traits and cognitive abilities should not be focusing our energies on what name adorns our banner. Our unique skill sets are all applied to asking meaningful mechanistic questions, whether behavioral, cognitive, neurobiological, or (almost certainly) some combination thereof, that advance our shared scientific aims. The words of the late Weisman (2008, p. 142) are particularly apt here: “Our job as natural scientists is to explain nature.”

The main thrust of Abramson's article is the question, “[W]here is the next generation of comparative psychologists going to come from?” While he suggests a number of excellent solutions for increasing undergraduate interest in comparative psychology, they seem largely fixes in search of a problem. In fact, the present authors have both used earlier versions of introductory texts that Abramson takes to task as failing to properly represent the field, and later were further exposed to our chosen field by mentors using texts (e.g., Schwartz, 1989) gallingly titled something other than “Comparative Psychology.” We represent different generations of comparative researchers tasked with training the next generation.

And perhaps the best solution to the search for comparative psychology undergraduates is telegraphed absently in the final paragraph of the essay, wherein Abramson discusses the use of “affiliated faculty” for assisting in teaching and advising comparative graduate programs. We no longer live in a scientific world of monolithic theoretical institutions, but rather one featuring a web of interconnected, specialist subfields. Papini (2011, p. 211) beautifully captured this idea in saying: “As a field, comparative psychology is almost interdisciplinary by definition…[and] demands not only knowledge of psychological theories and techniques, but an understanding of behavioral neuroscience, comparative neurology, behavioral ecology, developmental biology, and evolutionary theory.” This echoes the suggestion of Shettleworth (2009, p. 216) that “exploiting and nourishing those connections, and teaching our students to do the same, is an essential part of the future of the field.” The answer that thus seems obvious to us is that the next generation of comparative psychologists will come from the same constituent fields as the experts that comprise or complement the discipline, in perhaps only a slightly more skewed manner than they already always have.

## Comparative psychology is dead. long live comparative psychology!

We take no issue with the suggestions offered by Abramson, as they present reasonably straightforward ways of contacting students and researchers of disparate scientific backgrounds in the pursuit of expanding the reach of comparative research. Instead, we disagree with the concept that comparative psychology withers in obscurity when it is in fact composed of a number of thriving, interconnected subfields. The call to improve the offerings and uptake of comparative science should not just go out to those who deliberately call themselves comparative psychologists, but rather to those experts who never quite realized that they do in fact spend their lives pursuing comparative psychology.

For our part, we have no qualms about considering “comparative cognition” to simply be a subfield of “comparative psychology,” and we intuitively often conflate the two; many studies in animal cognition laboratories already hew close to interdisciplinary boundaries that do not necessarily include a heavy focus on cognition *per se*. The *Journal of Comparative Psychology* and *Frontiers: Comparative Psychology* are both eminent destinations for findings from comparative cognition and ethology laboratories. We all study similar evolutionary questions using similar behavior-based methods owing our history to the same pioneering scientists, with the singular exception that comparative cognition often seeks specifically to provide evidence for (and against!) particular mentalistic processes (Zentall, 2013). And surely both disciplines provide highly similar learning opportunities for students. Creating a false dichotomy only fractions a specialized field unnecessarily.

If comparative psychology is in crisis, we suggest it may be a crisis of identity rather than of continued existence. And rather than navel-gazing about how comparative psychologists can prevent the “absorption” of the field into others, it would be more constructive to see the field as an opportunity to bring together related sciences under an integrative, collaborative umbrella. Comparative psychology will continue to flourish so long as there are those who study behavior and cognition across species, and will continue to be useful so long as comparative researchers have these research themes to unify them.

## Funding

This work was supported by a Natural Sciences and Engineering Research Council of Canada (NSERC) Discovery Grant to CS.

### Conflict of interest statement

The authors declare that the research was conducted in the absence of any commercial or financial relationships that could be construed as a potential conflict of interest.
